# The meaning of adaptation in aging: insights from cellular senescence, epigenetic clocks and stem cell alterations

**DOI:** 10.1038/s43587-023-00447-5

**Published:** 2023-06-29

**Authors:** Mikolaj Ogrodnik, Vadim N. Gladyshev

**Affiliations:** 1 Ludwig Boltzmann Research Group Senescence and Healing of Wounds, Vienna, Austria; 2 Ludwig Boltzmann Institute for Experimental and Clinical Traumatology in AUVA Research Center, Vienna, Austria; 3 Austrian Cluster for Tissue Regeneration, Vienna, Austria; 4 Division of Genetics, Department of Medicine, Brigham and Women’s Hospital and Harvard Medical School, Boston, Massachusetts, US

## Abstract

With the recent rapid progress in the aging field, there is increasing evidence that many features commonly attributed to mechanisms/drivers of aging in fact represent adaptations. Here, we examine several such features, including cellular senescence, epigenetic aging and stem cell alterations. We draw a distinction between causes and consequences of aging and define short-term consequences as “responses” and long-term ones as “adaptations”. We also discuss “damaging adaptations”, which despite having beneficial effects short-term, lead to the exacerbation of the initial insult and acceleration of aging. Features commonly recognized as “basic mechanisms of the aging process” are critically examined here for the possibility of their adaptation-driven emergence from the processes like cell competition and wound-like appearance of the aging body. Finally, we speculate on the meaning of these interactions for the aging process and their relevance for the development of anti-aging interventions.

## Introduction

**Figure 1:**
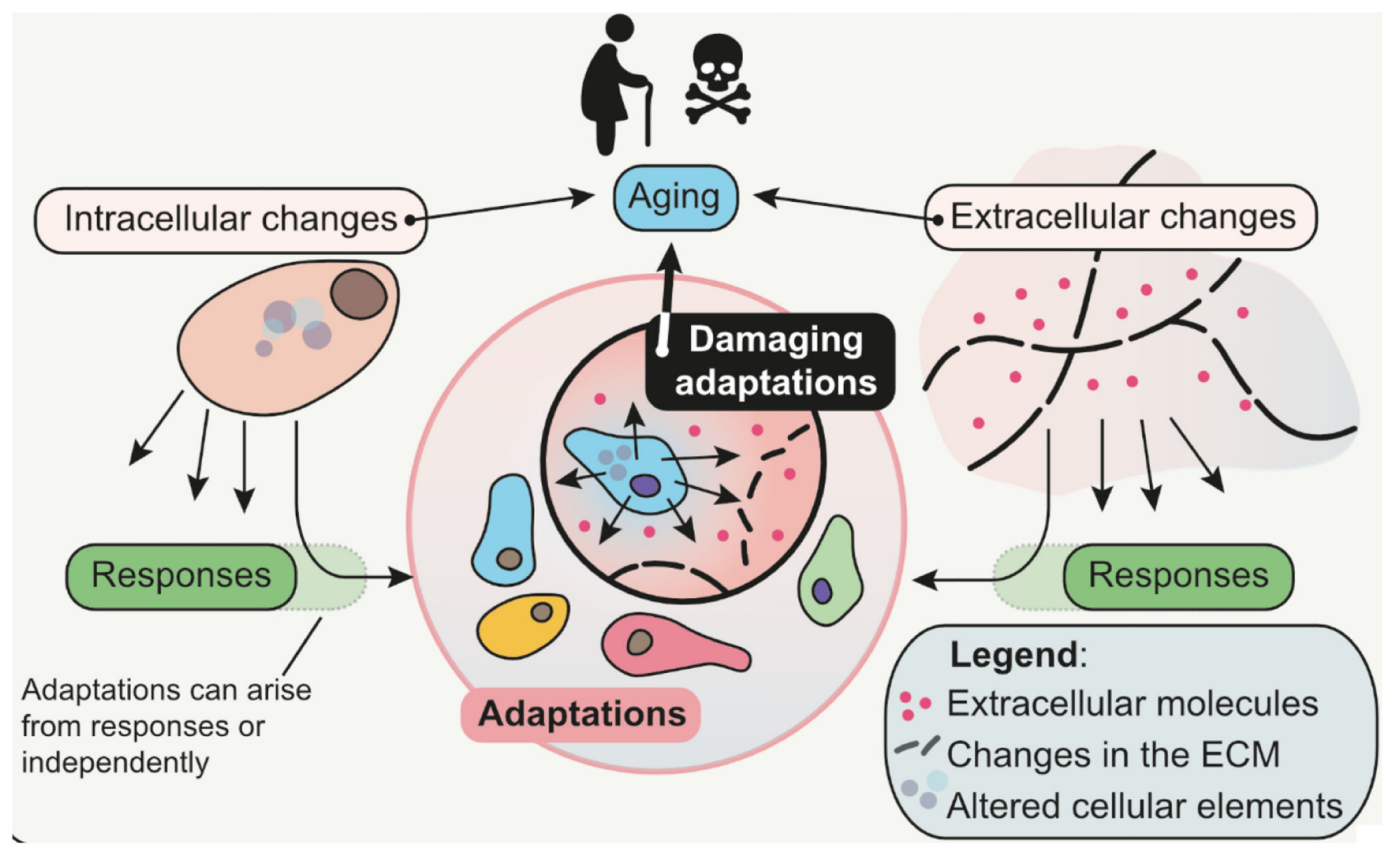
Relationships between causes and consequences of the aging process. Aging leads to changes at all levels of organismal complexity including damage accumulation, alterations of the extracellular matrix (ECM), inflammation etc. These and other changes lead to short-term responses and long-term adaptations of cells, allowing them to thrive and survive. A subset of these adaptations is “damaging”, i.e. with the negative consequences outweighing the benefits and leading in the long-term to the exacerbation of the aging process.

Aging is a highly complex biological process associated with a plethora of changes. However, not all changes observed in old tissues are necessarily drivers of the aging process or are even important for it. The distinction between causes and consequences of aging is especially relevant for designing interventions aimed at extending healthy lifespan. The reason is that targeting features that change with age but are not drivers of aging might be ineffective or even harmful. However, drawing the distinction between causes and consequences of aging is not easy. This is because many of the cellular and molecular phenotypes found in aged tissues represent adaptations of cells to their internal changes or the everchanging environment (Fig. 1). These adaptations, while downstream of the degenerative events that truly represent aging, themselves play a crucial role in the process. A comprehensive description of aging-related adaptations requires comprehensive understanding, which we dive into in this paper.

Since the beginning of cellular life, cells have been maximizing their chances of survival and propagation. The transition to multi-cellular life imposed spatiotemporal restrictions on the division rate, motility and function of individual cells to favor survival and propagation of cell communities/organisms. Thus, for cells hosted within a multi-cellular organism, survival is not only about nutrient availability and cell integrity but also about the proper response to microenvironmental cues. Failure to properly respond to signals from the microenvironment leads to the elimination of irresponsive cells. For example, neurons that do not manage to connect to their tissues of destination during development suffer deprivation of the nerve growth factor (synthesized by target tissues) and undergo apoptosis^1^, the fate of ~1/3 of all neurons within two weeks after birth^2^. In contrast, if a cell manages to become “independent” and resistant to signals from the microenvironment, the outcome is cancer, collapse of the whole multi-cellular system, and death. Thus, effective responsiveness to the local microenvironment is the basis for multi-cellular existence, for example with beta cells producing insulin in response to glucose, muscle cells contracting upon stimulation by nerves, immune cells becoming pro-inflammatory in response to foreign antigens, etc. When environmental cues are aberrant or missing, properly responding cells will not be able to function effectively. For example, for the effective uptake of glucose, cells need to be co-stimulated with insulin, and in diabetes the reduction in the release of insulin can lead to starvation of cells in an otherwise nutrient-full environment. However, cellular responses can also occur due to cell-internal changes. Skin cells exposed to excessive sunlight/UV show profound DNA damage and respond with apoptosis^3^. Other types of cells can respond to DNA damage with cell cycle arrest, usually a temporal one^4^. In this context, we define **“response”** as a short-term change of cellular phenotype to match signals from the microenvironment or from the inside of cells.

A long-term or a permanent change of cellular phenotype upon an internal or microenvironmental stimulation is defined here as **“adaptation”**. The microenvironmental drivers of adaptations include changes of the extracellular matrix (ECM), presence or absence of secretory factors (e.g. cytokines or hormones), electrical or mechanical stimuli, etc. For example, repeated/continuous stimulation of neurons leads to structural and biochemical changes in their synapses and the long-lasting increase in signal transmission^5^. Upon wounding, fibroblasts sense alterations in the local microenvironment, becoming pro-inflammatory and pro-migratory for a prolonged period of time^6^. The cell-internal drivers of adaptation include a wide range of structural changes of molecules, changes in their quantity, localization or properties, changes that are usually defined as “damage” such as mutations, epimutations, aggregates, certain post-translational modifications, macromolecular breaks, etc. (for more details see^7^). Damage and other cell-internal changes including fragmented mitochondria or a leaky nuclear envelope increase the risk of failed cell division and the overall risk of death^7–9^. In terms of coping with damage, a cell can be induced to undergo senescence (see later) or commit to differentiation^10^, in both cases preventing cell cycle progression. Thus, similar to adaptations stimulated by the microenvironment, adaptations originating from cell-internal changes are set to maximize survival of cells. As for the origin of such adaptations, they could be a direct consequence of the responses or be response-independent. For the prior, responses transition to adaptations when the stimuli are repetitive (an “aggregate” of responses) or of a prolonged duration, such as adaptations to cold, starvation etc. The latter could be due to stimuli which are below the threshold to trigger a response or do not have a dedicated machinery needed for an effective response and mild damage forms would fulfil these criteria^7,11^. Following this line of thought, one can also find a “grey zone” of processes, which are challenging to be assigned to binary categories of responses and adaptations. Especially, with the prolonged or an “aggregate” of responses it is currently unclear what the threshold for a response to develop into an adaptation is, and how such a transition looks like. In this perspective piece we will focus on the end points of these transitions, thus discuss adaptations that arise during aging.

While adaptations are generally set to maximize cell survival, some adaptations can have negative long-term consequences for their host. Based on that, we define “**damaging adaptations**” as a subset of adaptations that emerge during aging and further accelerate and/or exacerbate the aging process. In the following sections we will dissect three areas of extensive research in the field of aging and discuss what types of adaptations they represent and whether they are of internal origin or driven by the microenvironment.

## Cellular senescence

Cellular senescence is a cell cycle arrest that brings a variety of phenotypic changes to cells including a pro-inflammatory phenotype^12^. Senescence is commonly associated with aging, but its appearance has also been frequently attributed to healing, regeneration and development^10^. The first thing worth clarifying about senescence is whether this process is *just* a response or an adaptation, i.e. whether senescence *in vivo* represents a short-term or a long-term change. Most studies on senescent cells examine a snapshot of an organ’s physiology (e.g. using frozen or formalin-fixed piece of tissue) making it impossible to tell how long the cells currently positive for senescence markers have been in this state. Nonetheless, much evidence collected from *in vitro* studies as well as numerous cross-sectional *in vivo* studies have reported an elevated level of senescence markers persisting for a period of time, suggesting that senescence is long-term, and as such an adaptation^13^. In addition, while responses underlying senescence such as DNA damage response or others^12^ have their dedicated cellular machinery, cellular senescence as such is not definable by any specific protein and rather develops gradually as an indirect consequence to prolonged and/or multiple responses, once again matching the criteria of an adaptation. Although *in vitro* studies showed that senescent cells do not recover from a high load of damage, overexpression of oncogenes or prolonged cell culture, there is currently no evidence for the notion that senescence *in vivo* pertains a *permanent* cell cycle arrest.

It is a matter of ongoing debate whether cellular senescence observed in older organisms is caused by cell-intrinsic changes or the microenvironment. On the one hand, it is well-established that a variety of damage forms cause cellular senescence *in vitro* and *in vivo*^11^, suggesting that senescence is a cell-internal adaptation. On the other hand, appearance of senescent cells, for example during development and in wound healing, is tightly regulated in a spatiotemporal manner^10,14–16^, suggesting that senescence *in vivo* can be an adaptation caused by the microenvironment. There has been a number of hypotheses on the origin of cellular senescence in aging, including protection against cancer^17^, accumulation of damage incompatible with cell division^11^, high activity of cell expansion pathways^18^, a combination of both damage and expansion stimuli^19^, dysfunction of the immune system^20^, and a wound-like signature of aging organs^10^. The matter is, however, more complex as senescence can arise not only from cell-internal or non-cellular environmental stimuli, but can also be induced by other senescent cells in a paracrine manner^21,22^. Additionally, there is a “grey zone” for senescence induction as senescence can be triggered and maintained by reactive oxygen species (ROS)^23^, which can originate from cell-internal, environmental or paracrine triggers^24^.

With the evidence supporting both cell-intrinsic and microenvironmental-driven senescence induction, it is worth noting that they are not mutually exclusive and that there is also a positive feedback loop between cellular and extracellular factors that drive senescence. For example, inflammation and ECM damage contribute to upregulation of metabolic pathways and damage accumulation inside the cells^25,26^ and *vice versa*, damaged cells are more proteolytic and pro-inflammatory, leading to disruption of their microenvironment^10,12^. Thus, as in any other aspects of aging, both the dysfunctional microenvironment and cell-internal changes can drive adaptations of cells, including the induction of cellular senescence.

**Figure 2:**
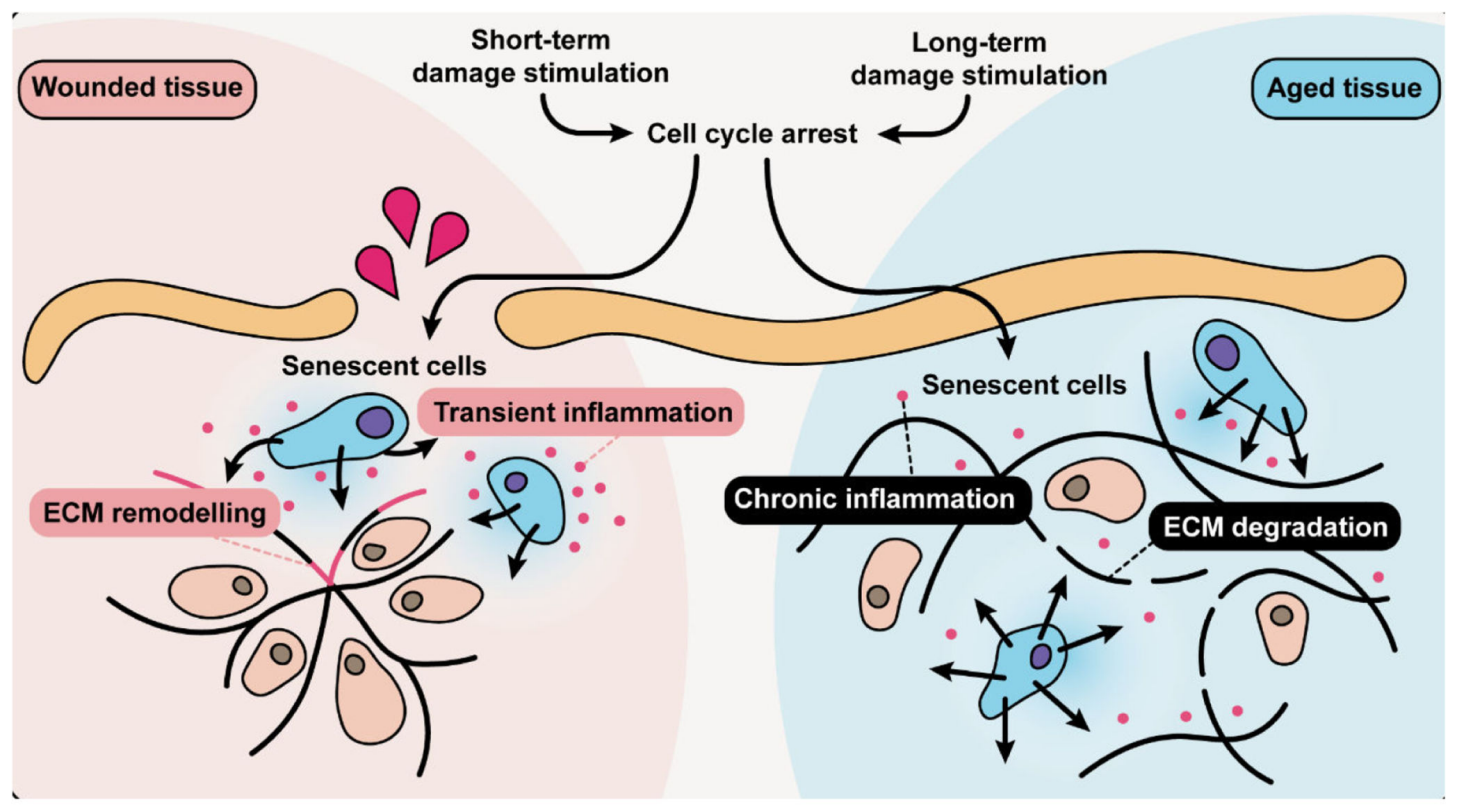
Cellular senescence as an adaptation to damage in tissues. A burst of damage that is a consequence of wounding results in an adaptive response, cell cycle arrest and induction of cellular senescence needed to cause transient inflammation and to remodel the extracellular matrix (ECM). Damage is also present during aging, but its irreparability leads to the persistence of cellular senescence, chronic inflammation and ECM degradation.

Another key question is whether this adaptation (senescence during aging) can be considered a “damaging adaptation”, and thus a promising target for anti-aging interventions. It might take many years of research and clinical trials before a reliable answer can be established, but the results reported so far are promising. It was reported that disabling the machinery required for cellular senescence induction can alleviate certain age-related dysfunctions; for example, mice with dysfunctional telomeres show improved tissue maintenance and survival following p21 depletion^27^. A number of studies in mice showed that the elimination of senescent cells is sufficient to alleviate a number of age-related diseases and to increase average lifespan^12,28^, albeit without a prominent effect on maximum lifespan^11,29^. However, there is increasing concern that the elimination of senescent cells could bring with it detrimental long-term consequences: recent research has revealed beneficial functions of senescence^10,14–16^, and elimination of p16-expressing senescent cells has been shown to lead to deterioration of health^30^. This pleiotropic role of senescence could be because the cell cycle arrest that underlies senescence is a response that has a context-specific function, with consequences varying between cell types and conditions (Fig. 2). For example, in health, cell cycle inhibitors are expressed when cells need to become more specialized, with arrested beta cells producing more insulin^31^, and arrested macrophages becoming more fit to fight off infections^32^. Inhibition of cell cycle is usually also coupled with the inhibition of cell death pathways, a feature prominent for both senescence and differentiation^10^. When it comes to aging, there are reports suggesting that cells acquiring features of senescence such as cell cycle arrest, for example neurons, do so in order to avoid death^10,33^. Interventions used to target senescent cells are not cell-type-specific (and often not senescence-specific) and the number of studies that investigated in detail what type of cells *are* and *are not* killed by senescence-targeting treatments is limited. Thus, more research is needed to assess the impact of senescence in aging and beyond to develop more selective anti-senescence interventions. Overall, senescence appears to be a type of damaging adaptation of aging cells resulting in an exacerbation of the aging phenotype.

## Epigenetic changes

DNA methylation at CG dinucleotides (CpG sites) is a type of epigenetic modification that has a number of functions, most notably the regulation of accessibility of transcription factors to the DNA^34,35^. A quantitative approach that relates age-associated changes in the DNA methylome with chronological age has been coined the “epigenetic aging clock” with the approximation of age it measures being called the “epigenetic age”^36^. Epigenetic clocks are now viewed as currently the best available tools for the estimation of biological age, however, the origin of epigenetic changes that underlies the clocks remains mysterious. One of the reasons why it is difficult to decipher the meaning of epigenetic clocks originates in the methodology behind their design. Essentially, epigenetic clocks are made by attributing a value to each relevant methylation site, such that multi-variate machine learning models enable a prediction of an individual’s age as accurately as possible. There are now multiple types of clocks designed to predict the age of tissues such as the blood^37^, liver^38^ or any tissue (multi-tissue)^39,40^ of mice and humans. There are also clocks that work with bulk samples and at a single cell level^41^, as well as clocks that are trained for phenotypic or functional changes, future mortality and the rate of aging^42–44^. The equations that these clocks are based on rely on different CpG sites and attribute different weights to them. In other words, clocks are not made to trace methylation sites of any specific genes, cell types, or biological processes, but rather are made so that the algorithm gives the most accurate age prediction, regardless of the origin or meaning of the variables it uses.

The DNA methylome consists of modifications that are often different between cell types and further change when cells execute specific functions^34^. Furthermore, some methylation patterns can be rapidly altered, for example during responses to metabolites, pathogens or other microenvironmental cues^45–48^, while other methylation patterns change slowly or persist, for example those responsible for cell identity or for supporting constitutive heterochromatin^34,35^. From this perspective, some methylome modifications would be “responses”, and others would be “adaptations”. When detected at the level of organs (e.g. in tissue lysates), the parameters used for the measurement of the epigenetic age of tissues might thus represent a mixture of reversible responses, long-term adaptations, alterations of gene expression, modifications of the accessibility to the non-coding regions of the DNA or changes in the proportions between populations of cells inhabiting a given tissue. One solution to distinguish cell-from population-level methylation changes would be with single-cell methylome sequencing^41^. However, even then, the diversity of information used to design the clocks is overwhelming, making it highly challenging to understand the role of clocks in aging and clarify whether epigenetic age could be considered a damaging adaptation.

Instead of investigating the clocks’ origin, let’s examine closely the biological phenomena for which the reliability of clocks has been tested. The clocks are designed to measure age-related changes, not only associated with “healthy aging”, but also with conditions known to accelerate aging such as obesity, smoking, trauma and certain genetic disorders^36^. Would epigenetic changes underpinning the clock models be driven by cell-internal changes or microenvironmental stimulations? In support of the latter, it was recently shown that some forms of damage such that DNA breaks do not increase the clock readout and that conditions leading to a rapid induction of senescence such as X-ray irradiation and overexpression of oncogenes do not increase epigenetic age^49^.

While a rapid induction of senescence does not elevate the predicted epigenetic age, a gradual senescence induction as in the case of replicative senescence does increase it^49^. Importantly, an increase in epigenetic age during replicative aging was not strictly related to the induction of cell senescence (i.e. conditions of late passages), but instead showed a gradual increase over time throughout the duration of cell culture^49^. Together with the observation that even immortal cells, e.g. telomerase reverse transcriptase (TERT)-expressing fibroblasts, show an increase in epigenetic age in cell culture over time, it can be assumed that epigenetic aging is caused, in part, by the progressive adaptations to the cell culture conditions^49^. Similar to unicellular organisms, primary as well as immortal cells maximize their survival and propagation chances while competing for resources and space in a cell culture dish. Such adaptations require changes in the DNA methylome and thus must be contributing to epigenetic aging.

Another causal factor behind progression of epigenetic clocks is differentiation. Not only less differentiated cells, such as those in the muscle and intestine^50,51^, exhibit younger epigenetic age than their more differentiated counterparts, but mixed populations of differentiated and non-differentiated cells show the epigenetic age matching the proportion between populations^49^. Similarly, differentiating retinal cells exhibit epigenetic aging^52^. Even more importantly, while undifferentiated stem cells do not show epigenetic aging, the process of epigenetic aging starts as soon as stem cells start differentiating^49^. These observations match the recent findings that the epigenetic clock in mice starts ticking around ~7.5E of development^41^, which roughly corresponds to the formation of three germ layers and the initiation of the differentiation processes, such as the transition of the inner cell mass to epiblast^34^ and epithelial-to-mesenchymal transitions^53^. Thus, the epigenetic age could be to a certain degree driven by the aging-induced progressive differentiation of cells^10^ and depletion of stem cells^54^. This, however, cannot be the sole source of aging-driven alterations of the methylome as epigenetic clocks also work very well for tissues that do not have many known stem cells, such as liver and blood. These changes in the methylome could be driven by systemic or microenvironmental adaptations as epigenetic aging has been shown to be influenced by the alterations and damage of the ECM, changes in nutrient availability and sensing, cellular metabolism and mitochondria biogenesis^40,47,49,55^.

While there is a lot of evidence that epigenetic clocks measure aging-induced microenvironmental adaptations, it remains unclear whether these adaptations, or more generally, age-related changes in the methylome, are damaging. In this respect, it was recently shown that a model of dysfunctional epigenetics shows accelerated aging and higher readouts of the epigenetic clocks^56^. However, as the model is not specific to epigenetic clock-related methylations, it is unclear whether clock-specific methylations could be considered “damaging”.

A recent study leveraged large-scale genetic data in combination with epigenome-wide Mendelian Randomization in an attempt to identify CpG sites causal to age-related traits, such as lifespan, healthspan and longevity^57^. It also developed a framework for integrating causal knowledge into epigenetic clock models that measure age-related damaging and adaptive changes. Interestingly, the former model was associated with various adverse conditions (e.g., mortality risk), whereas the latter was related to beneficial adaptations. Both clock models could be used to predict epigenetic age, but they performed differently in response to interventions^57^. This study further reinforces the idea of adaptive changes in the epigenome during aging.

**Figure 3:**
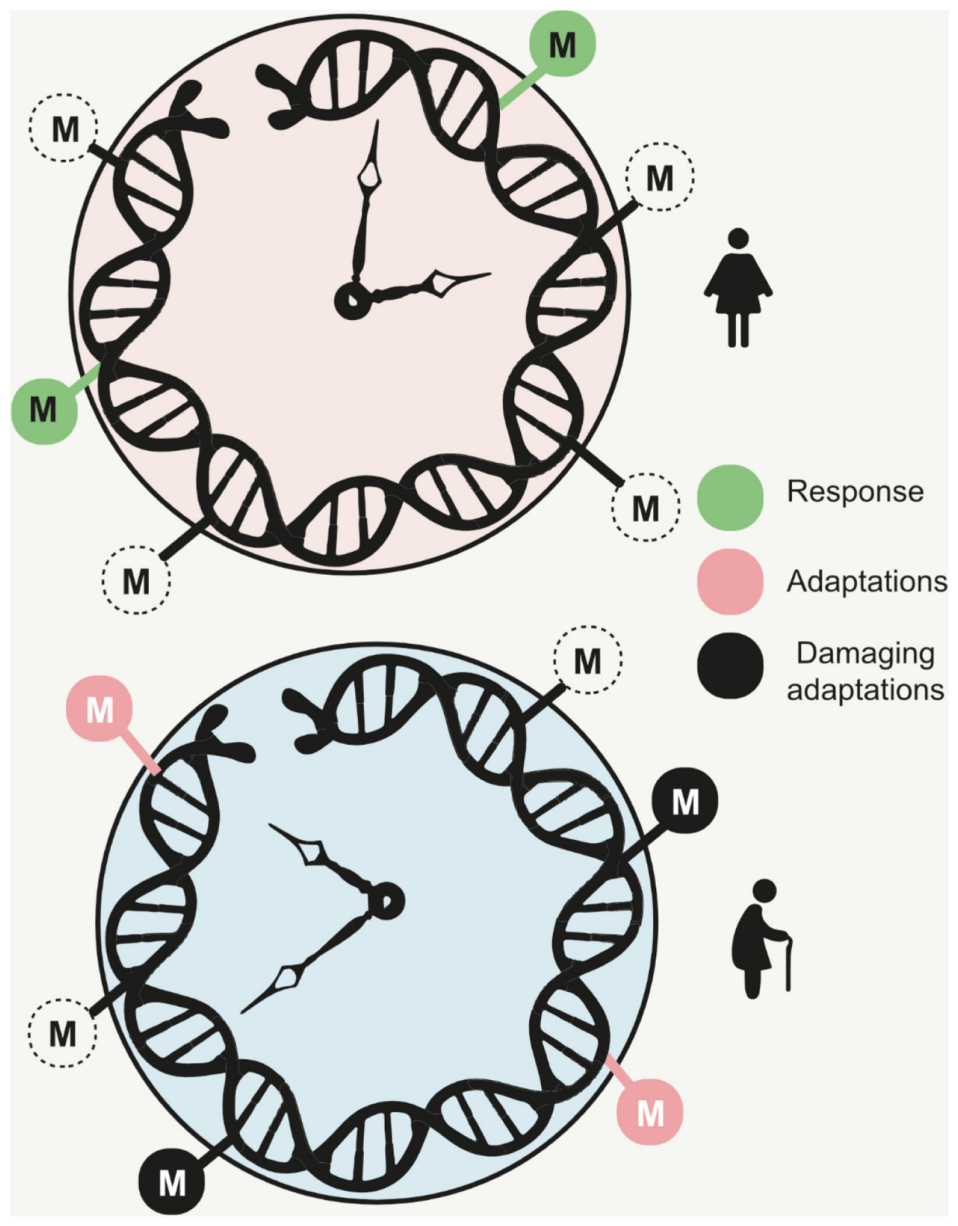
Age-related changes in DNA methylation are associated with diverse processes. Changes in DNA methylation status of CpG sites between young (upper) and old (lower) individuals may reflect short-term responses and long-term adaptations, including damaging adaptations. M is a methyl group. Open circles show CpGs that previously were methylated, and filled circles show currently methylated sites.

As the clocks are integrative biomarkers, it is likely that they include signals of both damaging and non-damaging adaptations (Fig. 3). This is consistent with the deconstruction of clocks resulting in differentially behaving modules^58^. However, further research is needed to deconvolute epigenetic clocks and identify their subsets representing diverse adaptations. While total epigenetic changes may maximize chances for survival of cells in the face of systemic and microenvironmental changes that underly aging, some of them may be the consequences of the other. Although being amazing tools to characterize and quantitively measure the process of aging, the diversity of epigenetic clocks and their complex composition make them unlikely targets for anti-aging interventions.

## Stem cell alterations

Numerous changes and dysfunctions of the stem cell compartment occur during aging, including skewing of the differentiation lineage of stem cells, a reduction in proliferation, and an overall decline in stem cell number^54^. Remarkably, these age-related deficiencies come as a surprise, as stem cells themselves seem to be more resistant to the aging process than other cell types. They have a high repair capacity^59,60^, an ability to regulate the length of their telomeres^61^ and robust mechanisms to remove an already accumulated damage via asymmetric distribution^62–64^. Empirically, at least for some types of stem cells the cell-internal consequences of the aging process, such as cell cycle arrest and DNA damage, are infrequent or even absent^65–67^. This disparity between the functionality and the state of stem cells in aged tissues can be explained by the dependency of stem cells on their microenvironment. Like no other cell type, stem cells strictly rely on their surroundings, called the “niche”. The microenvironment of stem cells does not only embed them within their tissue of origin, but also regulates their functionality including quiescence, proliferation rate, commitment towards the differentiation lineage and the responsiveness to systemic stimuli^54^. There is abundant experimental evidence supporting aging-induced degeneration of the niches, including an increase in their stiffness^68^, degeneration of the surrounding ECM^69–71^, a reduction in blood supply^72^, an increase in fat deposition^73^, an infiltration of immune cells and the consequent chronic inflammation^74^. We hypothesize that the majority of age-related changes in the function of stem cells are due to their adaptations to the conditions of aging niches. As the cellular machinery underlying adaptations originates from the cellular changes to the physiological stimuli, it would be safe to assume that there are certain conditions aside from aging where such adaptations are beneficial.

In addition to their role during tissue homeostasis, stem cells are extremely important during tissue injury, even to the extent that depletion of some types of stem cells has no effect on homeostasis, but impedes regeneration^75^. Skin stem cells, residing in hair follicles and in the basal layer of the epidermis, are mobilized out of their niches during wound healing in order to support re-epithelialization, among others^76,77^. It has been observed that in the aged skin, stem cells residing within hair follicles migrate to the epidermis, which has been causally linked to age-related hair loss^70^. As age-related changes of skin include an increase in inflammation and degradation of the ECM^78^, conditions that are highly resemblant of skin wounding^6^, it is possible that aged skin bears phenotypic resemblance to wounded skin^10^. Thus, the age-related changes in skin stem cells could be potentially attributed to their adaptations to the wound-like conditions of the aged skin.

Another example is the bone marrow providing a niche for hematopoietic stem cells (HSCs). These stem cells are responsible for the generation of new blood cells, both the red blood cells like erythrocytes originating in the process called “myelopoiesis” and the white blood cells like lymphocytes originating in the process of “lymphopoiesis”^54,79^. While the processes of myelopoiesis and lymphopoieses are balanced during homeostasis, certain physiological conditions can shift the balance towards the preference of one process over the other that skews the differentiation profile of HSCs. Such conditions include blood loss/hemorrhage when HSCs are stimulated to skew the differentiation profile with a concomitant increase in the generation of erythrocytes over lymphocytes^80^. In a surprising similarity, the aging process of the HSC compartment is well-known to result in the skewing of the differentiation process to generate fewer lymphoid and more myeloid progeny^79^. Knowing that the process of aging is associated with a general decline in the fitness and oxygen carrying capacity of erythrocytes^81–83^, it is possible that the commitment of HSCs towards myeloid differentiation is an adaptation compensating for the decrease in the functionality of erythrocytes in the aging body.

Finally, muscle stem cells (MuSCs) are known to be responsible for muscle regeneration in conditions of injury and minor damage originating from exercise^84,85^. While small injuries result in MuSCs differentiating into muscle cells, deep injuries cause muscle fibrosis^86^. Similarly, the aging process results in MuSCs being “primed” towards differentiation^87,88^, with a fraction of progeny becoming pro-fibrotic^89^. As the aging process of the muscle is accompanied by the degradation of the ECM and an increase in inflammation^71,90^, the aging-induced changes in MuSCs could originate from an injury-like state of the muscle tissue. In summary, aging-associated changes in stem cell function could derive from the degradation and inflammation of their niches resembling the conditions of the wounded tissues and leading to adaptations aimed at aiding their regeneration.

**Figure 4:**
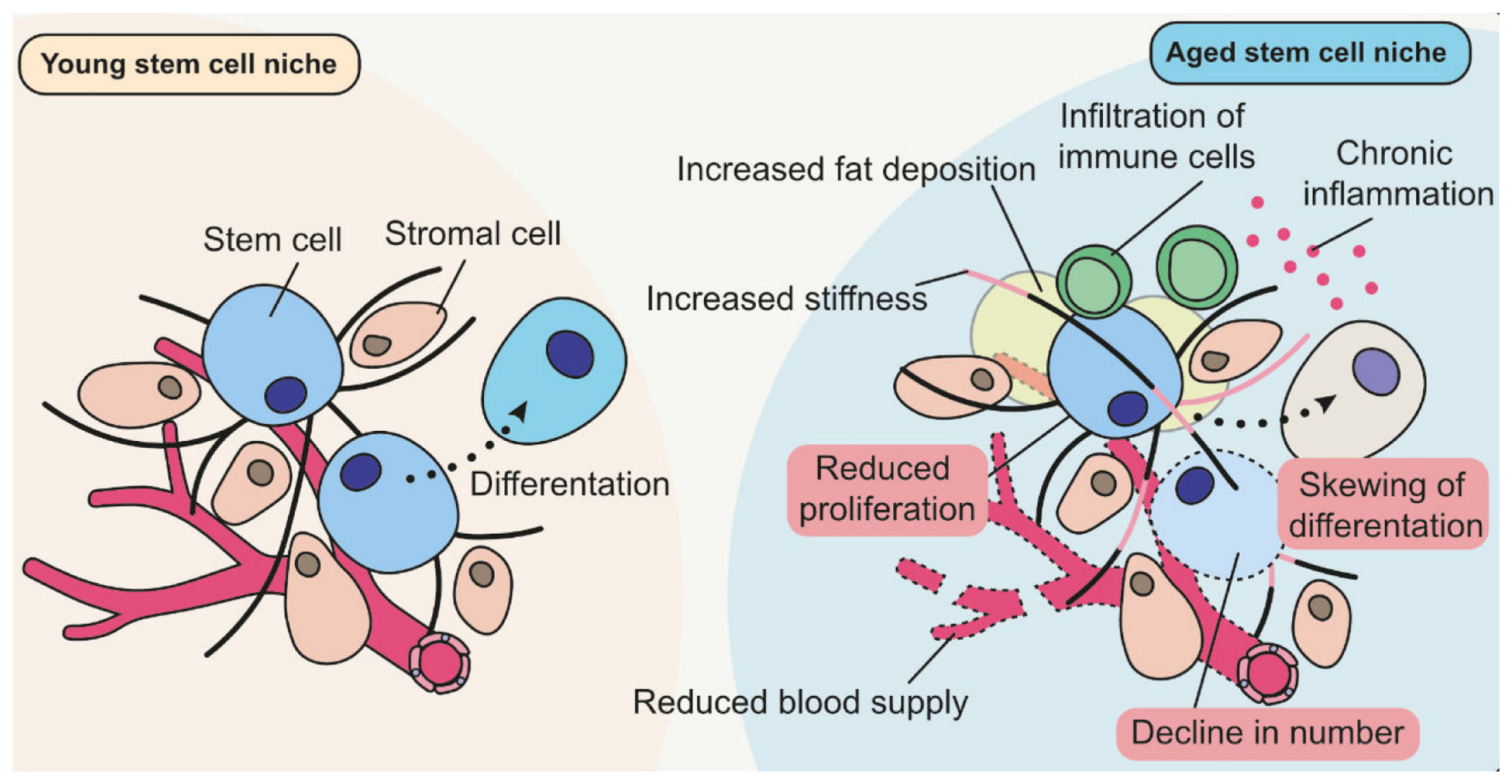
The age-associated alterations of stem cells are driven to a large extent by adaptations to a degrading niche. Adaptations of stem cells found in aged organisms are highlighted in red.

Another question is whether the aging-associated changes in stem cell function are a long-term adaptation or *just* a short-term response. Treatments such as parabiosis have proven that at least some of the age-induced changes in the stem cell compartment depend on the presence and absence of systemic factors and are reversible over a short period of treatment time^91,92^. This suggests that at least some aging-associated features of stem cells are direct responses to the current state of the body. However, many types of stem cell seem to be resistant to the presence of rejuvenating factors and/or reduction in pro-aging factors in the circulation^93^. Similarly, experiments involving transplantation of stem cells show that even when they are integrated into youthful niches, stem cells from aged animals retain certain aging-related features^68,94,95^, suggesting that the aging process has at least a long-term if not permanent impact on them. Overall, stem cells react to the aging process in a complex manner, with the short-term and reversible responses alongside the long-term adaptations (Fig. 4).

Finally, it is challenging to estimate how “damaging” these stem cell adaptations are to the aging process. Certainly, these adaptations have negative consequences, such as a decline in the propensity of HSCs to differentiate into lymphocytes, thereby increasing the risk of infection^96^, and the excessive migration of epidermal stem cells out of hair follicles, thereby causing hair loss^70^. However, it is not clear whether the alternatives are better. If stem cells were to be prevented from changing their phenotypes in relation to the wound-like conditions of the aged tissues would that bear beneficial effects or accelerate tissue degradation and aging even further? To illustrate, it has been hypothesized that the selection of mutant HSC clones makes them more pro-inflammatory^97^, which could potentially be beneficial for tissue regeneration or to counteract infections, but on the other hand these clones seem to exacerbate age-related conditions such as cardiac infarction^97^.

In summary, the aging process of the stem cell compartment appears to be tailored to the needs of the aging body. With a progressive degradation of the ECM and systemic cueing of tissue damage, stem cells in aged tissues are geared towards regeneration rather than homeostasis. Thus, it is difficult to assess whether treating aging-associated changes in stem cells would provide a robust target for anti-aging interventions.

## Linking it back together: adapt to survive at all costs

Each and every aspect of our lives, whether in health or disease, bears witness that our cells constantly strive to thrive and survive. It is thus rather unlikely that aging conditions would be an exception, where a malicious program sets in motion systems and means to drive disease and death. Instead, aging manifests as a progressive accumulation of intra- and extracellular damage and a consequential change in function in many types of cells that attempt to adapt and survive by any means necessary. Some of these means might work short-term, but in the long run they become damaging, and further exacerbate and accelerate the aging process.

The examples of adaptations to the aging process discussed above include cellular senescence, methylome alterations used to measure the epigenetic age, and changes in the stem cell compartment, but it is clear that such adaptations are widespread in other systems too. The strategies behind these adaptations are versatile, with the justification for cellular senescence being the most obvious, as the cell cycle arrest it imposes provides an alternative fate to cell death. When it comes to stem cells, there is evidence that their adaptations provide an advantage during aging-induced clonal selection in tissues such as the skin^98^, bone marrow^99^ and muscle^100^. Interestingly, it was shown that some of the genes positively selected include epigenetic regulators such as DNA methyltransferase 3A^101^. Despite some data suggesting that senescence does not accelerate epigenetic aging *in vitro*^49^, overall, senescence induction leads to prominent changes in the DNA methylome^102^, therefore it is possible that both these adaptations could contribute to epigenetic aging as measured by the epigenetic clocks. Another element positively selected for the survival of stem cells during aging is their attachment to the matrix. While during aging there is a decline in the ECM supporting the niche, skin stem cells that show a compensatory increase in expression of ECM proteins^103^ are better attached to the ECM and thus are selected for survival^69^. Similarly, genes of the MuSCs positively selected during aging are responsible for integrin signaling^71^ and other regulators of stem cell-niche interactions^50^. Senescent cells are not only strongly attached to the surface^104^, but also tend to inhibit the detachment and death of the surrounding cells^105^. While preserving lives of cells, this paracrine effect of senescent cells can be tumorigenic^105^, thus negative long-term. Overall, the experimental evidence suggests that cells of aging organisms employ any means necessary to avoid death including niche-driven alterations of stem cells and senescence induction of differentiated and progenitor cells.

In this piece, we use the term “adaptations” in relation to events happening within a single lifespan of an organism, in contrast to “evolutionary adaptations” that drive changes across generations of organisms enabling them to thrive in a given environment. Nonetheless, the processes described here may be complementary to the ideas on evolution of aging, e.g. postulates of the Medawar’s and Williams’s evolutionary theories^106,107^. Briefly, Medawar proposed that organisms evolved features that are neutral early in life while selection is actively taking place, but are detrimental later, where they become unfixable by evolution^106,107^. Going one step further, Williams hypothesized that evolution selects for features providing benefits early in life even if these features have negative consequences late in life. Likewise, we argue that some adaptations while increasing survival over the course of lifespan contribute to aging in the long-term. Also, integral components of the three types of adaptations we described above can be found throughout the early life of an organism: senescent cells are present during development and healing, epigenetic clocks tick during these processes, and stem cells show phenotypic similarities during the processes of regeneration and aging. Even when beneficial in early life, these phenotypes can become damaging later on, in line with the Williams’s concept of antagonistic pleiotropy.

## The matter of causality and targeted interventions in aging

While the identification of causal factors is straightforward for processes such as wound healing and fighting off infection, aging is multi-factorial and presents a landscape of intra- and extracellular causes in the context of responses and adaptations of cells. With the dense network of interactions between aging cells and their host tissues it is challenging to distinguish what comes first. In this respect, we offer the perspective that features of aging assigned as adaptations cannot be the primary drivers of the aging process, but some of them may nonetheless exacerbate the aging process. This perspective further assumes that the adaptations found in aging are not there to drive the aging process, but are an attempt to alleviate age-related changes regardless of whether this is possible or not (i.e. pro-healing activation of cells due to the wound-like appearance of an aged tissue is unlikely to actually heal it). Finally, the category of “damaging adaptations” describes adaptations which are predominantly detrimental to the aging process, making them a tempting target for anti-aging interventions. However, as the processes underlying “damaging adaptations” are not specific to aging, targeting them could have side effects for conditions where such adaptations are beneficial (such as tissue regeneration). Moreover, targeting these adaptations is unlikely to have a major effect on the aging process itself, as the underlying processes are not going to be affected.

Ideally, we would want to target the very basal and specific causal factors behind the aging process, as there could be no side effects coming from their selective targeting. While the current progress of the science of biogerontology seems promising when it comes to defining a variety of adaptations to the aging process such as senescence, stem cell alterations and methylome changes, much less is known about the processes causal to adaptations, and the science behind these processes is still in its infancy. The variety of mild damage forms accumulating in cells and in the ECM and likely other molecular processes are mostly beyond the detection limit, without any possibility of specific targeting. In this respect, we encourage scientists to expand the technical, conceptual and experimental scope of their research to pioneer the science of the drivers of aging.

## Conclusions

The three exemplary adaptations described here were intentionally selected to be highly diverse and seemingly unrelated. The conceptual framework we created here is aimed at showing that essentially any of the prominent targets of aging research and anti-aging interventions can be dissociated into sets of phenotypes representing responses and adaptations to the cell-internal and external changes occurring during aging. However, even if secondary to the basal processes of aging and set for survival of individual cells, these adaptations might cause more damage that exacerbate the initial insult. While each one of such damaging adaptations appears to be a promising target for anti-aging treatments, at this point it is not obvious which of these are “sufficiently” damaging that their alteration or elimination would be considered beneficial long-term. With the advent of single-cell omics, advanced genetic tools and high-resolution, multi-target spatial histology, it becomes more realistic to spatiotemporally map the plethora of changes occurring during the aging process and establish their long-term consequences, thus shedding light on the causal factors in aging providing the most optimal therapeutic targets.
